# Mapping the frontiers of genomic disease surveillance research in the ASEAN region: scientometric insights for precision public health and next-generation epidemic intelligence

**DOI:** 10.1093/bib/bbag573

**Published:** 2026-10-15

**Authors:** Jerico Bautista Ogaya, Rahmatullah Nazari, Olalekan John Okesanya, Andres Phillip Mayol, Emmanueal M Cabanig, Mohamed Mustaf Ahmed, Murtaza Jafari, Roel Nickelson M Solano, Joey Salinas Ramos, Zhinya Kawa Othman, Adriana Viola Miranda, Shuaibu Saidu Musa, Mae Frances G Sanico, Rolando A Alimen, Lin Xu, Junjie Huang, Thijs Kouwenhoven, Yusuff Adebayo Adebisi, Don Eliseo Lucero-Prisno

**Affiliations:** Department of Medical Technology, Institute of Health Sciences and Nursing, Far Eastern University, Nicanor Reyes Street, Sampaloc, Manila, 1015, Philippines; Center for University Research, University of Makati, J.P. Rizal Extension, West Rembo, Taguig City, Metro Manila, 1644, Philippines; Medical Science Research Center, Asas Medical University, Hissa-e-Duwum-e-Khair Khana, between Qala-e-Najarha intersection and Gulai Cinema, Kabul, 1008, Afghanistan; Faculty of Medicine, Department of Public Health and Maritime Transport, University of Thessaly, Argonafton & Filellinon, Volos, 38221, Greece; Department of Medical Laboratory Science, Neuropsychiatric Hospital, Aro, P.M.B. 2002, Abeokuta, Ogun State, 110001, Nigeria; Department of Biomedical Manufacturing, and Robotics Engineering, De La Salle University, 2401 Taft Ave, Malate, Manila, 1004, Philippines; Center for Engineering and Sustainable Development Research, De La Salle University, 2401 Taft Ave, Malate, Manila, 1004, Philippines; Department of Computer Engineering, Southern Leyte State University, Sogod, Southern Leyte, 6606, Philippines; Department of Data Science, SIMAD University, Jidka Warshadaha 2526, Wadajir District, Mogadishu, Banadir Region, Somalia; School of Global Health, Faculty of Medicine, Chulalongkorn University, 1873 Rama IV Rd, Pathum Wan, Bangkok, 10330, Thailand; Medical Research Center, Shifa University, Kote Sangi Main Road, Company, 5th District, Kabul, 100521, Afghanistan; Department of Medical Technology, College of Health Sciences Education, University of Mindanao, Matina Campus, McArthur Highway, Davao City, 8000, Philippines; Department of Medical Technology, College of Health Sciences Education, University of Mindanao, Matina Campus, McArthur Highway, Davao City, 8000, Philippines; Faculty of Pharmaceutical Sciences, Chulalongkorn University, PGVJ+7G7, Pathum Wan, Bangkok, 10330, Thailand; Department of Research, Global Health Focus Asia, Bandung, West Java, 40115, Indonesia; School of Global Health, Faculty of Medicine, Chulalongkorn University, 1873 Rama IV Rd, Pathum Wan, Bangkok, 10330, Thailand; Department of Nursing Science, Ahmadu Bello University, Sokoto Road, Samaru, P.M.B. 1045, Zaria, Kaduna State, 810211, Nigeria; Research Services Office, Palompon Institute of Technology, Evangelista Street, Palompon, Leyte, 6538, Philippines; Research and Extension Office, John B. Lacson Foundation Maritime University, M.H. del Pilar Street, Molo, Iloilo City, 5000, Philippines; Department of Thoracic Surgery, the First Affiliated Hospital, School of Medicine, Zhejiang University, 1367 Wenyi West Road, Yuhang District, Hangzhou, Zhejiang, China; The Jockey Club School of Public Health and Primary Care, Faculty of Medicine, Chinese University of Hong Kong, Ma Liu Shui, New Territories, Hong Kong SAR, China; Centre for Health Education and Health Promotion, Faculty of Medicine, Chinese University of Hong Kong, Ma Liu Shui, New Territories, Hong Kong SAR, China; Department of Physics, Xi’an Jiaotong-Liverpool University, Wuzhong District, Suzhou, 215000, China; Department of Epidemiology, University of Glasgow, Glasgow, G12 8QQ, United Kingdom; Department of Global Health and Development, London School of Hygiene and Tropical Medicine, Keppel St, London, WC1E 7HT, United Kingdom; Research and Development, Kalinga State University, Bulanao, Tabuk City, Kalinga, 3800, Philippines; Research, Extension, and Innovation, Pangasinan State University, Alvear E, Poblacion, Lingayen, 2401, Pangasinan, Philippines

**Keywords:** pathogen genomics, infectious disease surveillance, precision public health, epidemic intelligence, ASEAN, scientometric analysis

## Abstract

Genomic disease surveillance has become a cornerstone of precision public health, yet the regional research architecture in Southeast Asia remains insufficiently characterized. This study provides a comprehensive scientometric mapping of genomic surveillance research in the Association of Southeast Asian Nations (ASEAN) region and identifies patterns relevant to the development of epidemic intelligence systems. A scientometric analysis of 8129 Scopus-indexed publications (2000–25) was conducted using Bibliometrix/Biblioshiny and VOSviewer software. Analyses included publication trends, citation patterns, collaboration networks, institutional productivity, funding sources, keyword co-occurrence, and thematic evolution. The field demonstrates robust annual growth (14.49%) and high international collaboration (43.92%). Thailand, led by Mahidol and Chulalongkorn Universities, emerged as the principal regional knowledge hub in terms of indexed scholarly visibility. Publication influence is concentrated in a limited set of high-impact journals (PLOS One, Emerging Infectious Diseases, Journal of Clinical Microbiology) and internationally connected author networks. Thematic evolution reveals a transition from molecular epidemiology toward whole-genome sequencing (WGS), antimicrobial resistance (AMR), zoonotic surveillance, and One Health intelligence. Growth modeling suggests sustained expansion through the early 2040s. Genomic disease surveillance research in Southeast Asia has grown substantially, with expanding international collaboration and thematic shifts toward WGS, AMR, zoonotic surveillance, and One Health. However, research output remains concentrated in a few countries and institutions, and bibliometric findings do not measure surveillance capacity or policy implementation. Further research integrating laboratory, funding, epidemiological, and policy evidence is needed across ASEAN.

## Introduction

Genomic disease surveillance is transforming the public health approach to infectious diseases by enabling high-resolution tracking of pathogens through advances in sequencing technologies and bioinformatics [1]. The integration of whole-genome sequencing (WGS) and next-generation sequencing (NGS) into public health systems has provided unprecedented insights into transmission dynamics, pathogen evolution, and outbreak detection [2]. These innovations underpin the emerging paradigm of precision public health, where interventions are increasingly informed by granular genomic data to enhance both effectiveness and timeliness [2, 3].

Globally, genomic surveillance has proven crucial in responding to major infectious disease threats, including influenza, Ebola, and most recently, Coronavirus Disease 2019 (COVID-19) [4]. Real-time genomic epidemiology during the COVID-19 pandemic rapidly identified hundreds of separate viral introductions and reconstructed transmission chains, directly informing public health decision-making and the national response [5]. Consequently, genomic surveillance is now recognized as a core component of next-generation epidemic intelligence systems, which integrate real-time data streams with advanced analytics to anticipate and mitigate health threats [6].

Southeast Asia represents a particularly critical region for genomic disease surveillance due to its unique epidemiological and ecological context. The region is characterized by high biodiversity, dense population centers, extensive human–animal interfaces, and significant cross-border mobility, all of which contribute to the emergence and spread of infectious diseases [7]. Historical outbreaks of Severe Acute Respiratory Syndrome (SARS), avian influenza (H5N1), and dengue have repeatedly underscored the region’s vulnerability to emerging pathogens and the necessity for robust surveillance systems [8]. Despite these shared challenges, Southeast Asia exhibits considerable heterogeneity in genomic capacity, infrastructure, and research output. While countries such as Thailand and Singapore have developed advanced genomic capabilities, others face persistent constraints related to funding, technical expertise, and data governance [9]. This uneven landscape raises important questions regarding the distribution of scientific knowledge, collaboration networks, and thematic priorities within the region. Although the global importance of genomic surveillance is well established, there remains a lack of systematic understanding of how research in this field has evolved within Southeast Asia. Specifically, gaps persist in identifying dominant research actors, institutional networks, thematic trajectories, and structural inequalities in knowledge production. Addressing these gaps is essential for informing regional strategies aimed at strengthening genomic surveillance systems and advancing health security.

A bibliometric analysis of 1989 Scopus-indexed antimicrobial resistance (AMR) publications from Southeast Asia (2014–24) identified four co-citation clusters, two of which were explicitly genomic: “Genomic intelligence in AMR: tools for resistance gene discovery” and “Data-driven phylogenomics and AMR evolution mapping” [10]. That study documented a steady rise in regional AMR output, peaking at over 320 articles in 2024, with genomic approaches becoming increasingly central over time. However, it was restricted to AMR and did not examine genomic disease surveillance as a distinct field, nor did it analyze regional collaboration networks, institutional concentration, or thematic evolution beyond resistance. A broader scientometric analysis of 12 511 infectious disease publications from Association of Southeast Asian Nations (ASEAN) countries (1925–2022) documented a significant increase in regional research output since 2003, with 76.91% of publications appearing in the last decade [11]. That study found that Thailand, Malaysia, and Singapore were the most productive countries, while research trends shifted from viral diseases and diagnostic approaches involving Polymerase Chain Reaction (PCR), gene, and sequencing techniques toward bacterial infections, herbal medicine, and alternative medicine. However, it examined infectious disease research broadly rather than genomic disease surveillance specifically, and therefore did not characterize collaboration networks, institutional concentration, or thematic evolution specifically within the genomic disease surveillance domain. Country-level and regional capacity assessments have documented pathogen genomics infrastructure in lower-resource Asian settings [12], but these are survey-based, cross-sectional, and do not trace long-term publication trends or cover all ASEAN member states. In addition, no bibliometric study has yet mapped the full ASEAN-wide genomic disease surveillance landscape from 2000 to 2025. Our study addresses this gap by analyzing 8129 documents from 1608 sources, covering publication growth, collaboration networks, thematic evolution, and institutional concentration across all ASEAN member states. This extends prior regional work in three ways: temporally (25 years versus 10 years), thematically (genomic disease surveillance broadly rather than AMR alone), and structurally (network and thematic evolution analysis versus descriptive trends).

Therefore, this study aims to conduct a comprehensive bibliometric mapping of genomic disease surveillance research in Southeast Asia. The objectives are to analyze publication trends and growth dynamics, identify key authors, institutions, and sources, examine collaboration networks, map thematic evolution, and generate strategic insights for advancing precision public health and next-generation epidemic intelligence in the region.

## Materials and methods

### Study design

This study employed a scientometric design to examine the structural evolution, collaborative networks, and thematic architecture of genomic disease surveillance research in Southeast Asia. Scientometric methods combine bibliometric performance analysis with network-based science mapping to assess the intellectual, social, and conceptual organization of a research field. By integrating descriptive metrics with co-authorship, keyword co-occurrence, bibliographic coupling, and thematic evolution analyses, the approach identifies growth trajectories, knowledge diffusion, institutional concentration, and emerging research frontiers over time. The framework was structured to capture macro-level productivity patterns alongside meso- and micro-level relational dynamics within the regional research ecosystem. Genomic disease surveillance was operationally defined as the use of genomic and molecular tools, including WGS, NGS, phylogenetics, and pathogen genomics, for the detection, monitoring, and epidemiological tracking of infectious diseases, consistent with contemporary precision public health paradigms.

### Data source

The bibliographic dataset was retrieved exclusively from Scopus (Elsevier), selected for its broad coverage of peer-reviewed biomedical, genomic, and public health literature and its standardized metadata architecture. Scopus is widely used in scientometric research, particularly for interdisciplinary fields such as genomic epidemiology that span clinical medicine, molecular biology, and health systems science. Compared with alternative databases, Scopus offers stronger representation of regional and applied research outputs, including studies from Southeast Asia, while maintaining consistency in author profiles, institutional affiliations, and citation tracking essential for reliable network construction and reproducible analyses. Although a single-database approach may underrepresent non-indexed or locally disseminated studies, particularly from lower-resource ASEAN settings, it prioritizes data integrity and comparability. Accordingly, observed disparities should be interpreted as differences in indexed scientific visibility rather than absolute research activity.

### Data search and extraction

A systematic data search was performed in Scopus database on 20 April 2026. To maximize retrieval efficiency while ensuring conceptual specificity and regional relevance, we developed a structured search strategy (Fig. 1). We queried the Scopus title, abstract, and keyword (TITLE-ABS-KEY) fields using validated terms spanning three primary domains: genomic technologies (e.g. WGS, NGS, pathogen genomics, phylogenetics), surveillance frameworks (e.g. disease surveillance, biosurveillance, outbreak detection, transmission dynamics), and geographic identifiers for all ASEAN member states and regional actors (Brunei Darussalam, Cambodia, Indonesia, Lao PDR, Malaysia, Myanmar, the Philippines, Singapore, Thailand, Vietnam, and Timor-Leste; see Supplementary Appendix SI).

**Figure 1:**
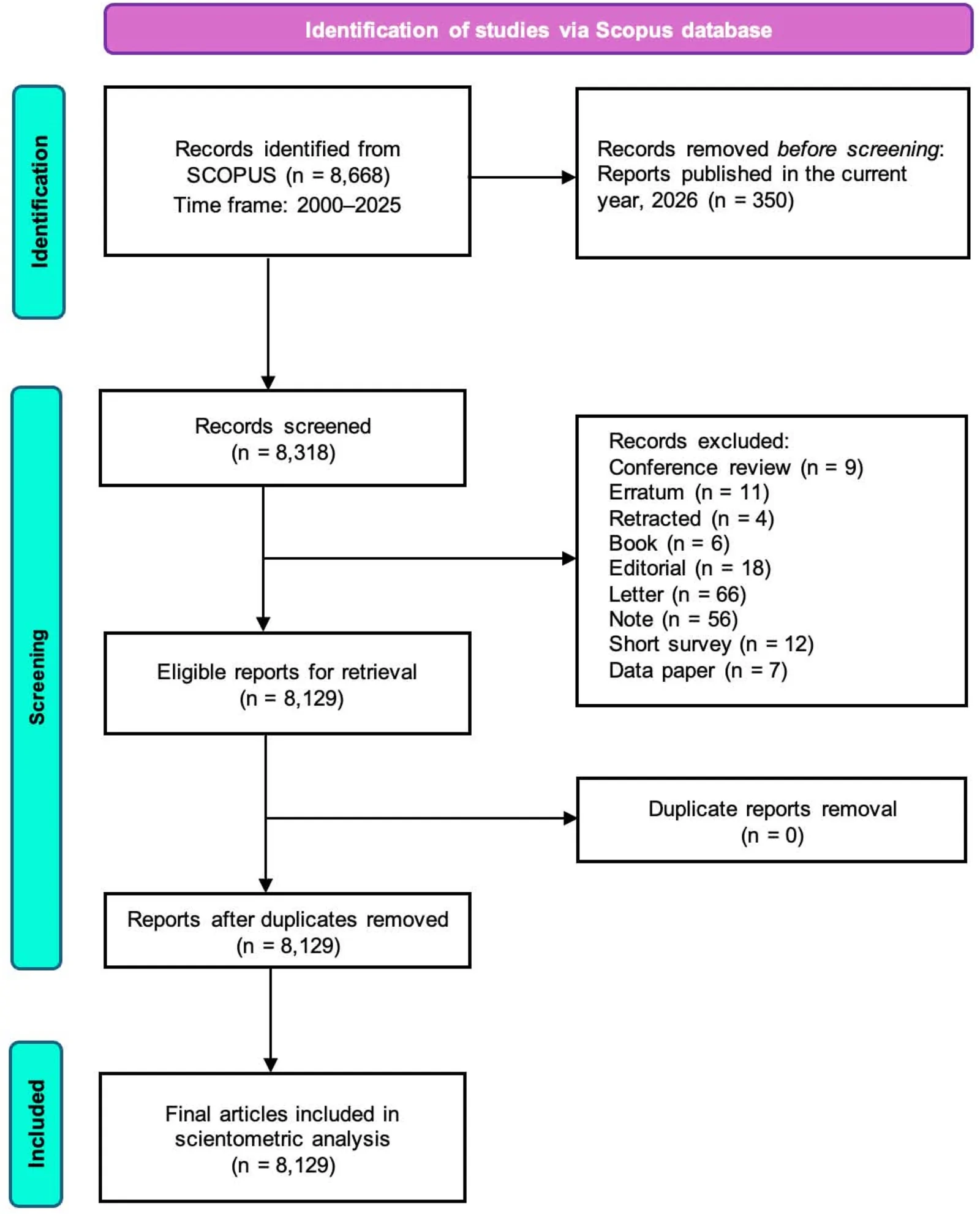
PRISMA flow diagram of the document retrieval and screening process for ASEAN genomic disease surveillance research.

To ensure temporal consistency and citation stability, the analysis was restricted to publications from 2000 to 2025, with records published in 2026 excluded because of incomplete citation accumulation and ongoing indexing. Eligible document types comprised peer-reviewed articles, reviews, conference papers, and book chapters, thereby capturing both established research and emerging contributions disseminated through conference proceedings. Conference papers were explicitly retained because conference-based dissemination represents an important component of research communication in bioinformatics, computational biology, genomics, and related rapidly evolving fields. In contrast, records classified by Scopus as conference reviews were excluded because they represented secondary or review-type conference contributions rather than primary conference research outputs and could introduce heterogeneity in the document corpus and potential duplication of evidence already represented in peer-reviewed review literature. Other non-research publication types, including errata, retracted records, books, editorials, letters, notes, short surveys, and data papers, were excluded according to the predefined eligibility criteria.

The analysis was restricted to English-language publications indexed in Scopus. English was selected to maintain linguistic consistency in title, abstract, and keyword-based retrieval and subsequent text-based scientometric analyses, particularly keyword normalization, co-occurrence analysis, thematic mapping, and conceptual clustering. This criterion also supported reproducibility and minimized potential inconsistencies arising from translation or language-dependent interpretation of technical terminology across a multilingual corpus. The restriction was applied as a methodological standardization criterion rather than as an assessment of the scientific quality of non-English publications.

### Data pre-processing and cleaning

Data preprocessing was conducted to ensure the accuracy, consistency, and analytical validity of the bibliographic dataset. This process involved systematic deduplication of records, removal of incomplete entries, and standardization of author names and institutional affiliations to address inconsistencies arising from indexing variations. Keyword harmonization was performed through the development of a manually curated thesaurus file, consolidating synonyms, spelling variations, and semantically related terms (e.g. “WGS” and “whole genome sequencing,” “COVID-19,” and “SARS-CoV-2”). This step was critical to improving the accuracy of co-occurrence networks and thematic clustering.

In addition, manual screening of titles and abstracts was undertaken to exclude irrelevant records that did not align with genomic surveillance or Southeast Asian contexts despite keyword overlap. This ensured thematic precision and minimized database noise. To enhance interpretability and reduce network fragmentation, minimum occurrence thresholds were applied across analyses, including ≥10 occurrences for keywords and country outputs. These thresholds facilitated the identification of significant patterns while minimizing the influence of sparse or peripheral data points.

### Validation of search strategy

The search strategy was developed through an iterative refinement process to maximize conceptual coverage while maintaining specificity to the study objectives. Candidate search terms were identified from relevant literature, controlled vocabulary, and commonly used terminology within the field, then refined through repeated trial searches to evaluate the relevance and consistency of the retrieved records. The final search query was subsequently examined through manual inspection of representative records and cross-validation against well-established publications identified from authoritative reviews and citation tracking to ensure adequate coverage of the core literature. Minor modifications were implemented to improve the balance between sensitivity and specificity while minimizing the retrieval of irrelevant records. Although no database search strategy can completely eliminate false-positive or false-negative retrievals, these validation procedures support the suitability of the final search query for comprehensive bibliometric analysis. Consequently, the findings should be interpreted as representing the indexed scholarly landscape captured by Scopus rather than the entirety of global research activity.

### Data analysis

Bibliometric performance and science mapping analyses were conducted using Bibliometrix/Biblioshiny in R and VOSviewer software (version 1.6.20.) Bibliometrix was used to generate descriptive and performance indicators, including annual publication trends, citation patterns, leading authors, productive sources, institutions, and countries [13]. VOSviewer was used to construct and visualize bibliometric networks using association strength normalization and clustering algorithms [14]. All analyses were conducted in R version 4.3.0 using the bibliometrix package (version 4.2.1) and VOSviewer version 1.6.20. The complete analysis code, cleaned datasets, package dependencies, and figure-generation scripts are available at (https://doi.org/10.5281/zenodo.20822849). The full search query is provided in Supplementary Appendix I. The detailed procedures, keyword harmonization dictionary, and geographic exclusion dictionary are available via the Zenodo repository at the DOI provided above.

Collaboration structures were examined at the author, institutional, and country levels to assess scientific cooperation, network centrality, and regional connectivity. Conceptual and intellectual structures were explored through keyword co-occurrence analysis, multiple correspondence analysis, bibliographic coupling, and co-citation analysis. A minimum threshold of ˃10 occurrences was applied across network analyses to reduce noise and strengthen interpretability. Overlay visualization was further used to identify temporal shifts, emerging research fronts, and evolving thematic priorities. Collectively, these analyses provided a multidimensional assessment of productivity, collaboration, and thematic evolution in genomic disease surveillance research across Southeast Asia.

## Results

Table 1 provides a comprehensive overview of the Scopus bibliographic metadata analyzed in this study, spanning a 25-year period from 2000 to 2025. The dataset comprises 8129 documents sourced from 1608 distinct outlets, reflecting an annual growth rate of 14.49%. The research landscape is characterized by its relative recency, with an average document age of 7.84 years and a mean of 27.1 citations per document. The data highlights a significant emphasis on collaborative scholarship; while the study involves 42 768 authors, only 118 documents are single-authored, yielding a high average of 8.9 co-authors per document and an international collaboration rate of 43.92%. In terms of content indexing, the metadata includes 13 865 author keywords and 26 198 Keywords Plus terms. Finally, the distribution of document types is heavily weighted toward primary research, consisting of 7524 articles, 382 reviews, 148 conference papers, and 75 book chapters.

**Table 1:** Overview of Scopus bibliographic metadata.

Description	Results
Main information about data	
Timespan	2000:2025
Sources (Journals, Books, etc.)	1608
Documents	8129
Annual growth rate %	14.49
Document average age	7.84
Average citations per doc	27.1
References	316 438
Document contents	
Keywords Plus (ID)	26 198
Author’s keywords (DE)	13 865
Authors	
Authors	42 768
Authors of single-authored docs	109
Authors collaboration	
Single-authored docs	118
Co-authors per doc	8.9
International co-authorships %	43.92
Document types	
Article	7524
Book chapter	75
Conference paper	148
Review	382

### Research productivity and temporal dynamics

The longitudinal data from 2000 to 2025 demonstrates an exponential expansion in ASEAN genomic disease surveillance research, with annual output increasing by ~2848% from a baseline of 33 publications (Fig. 2). This trajectory can be differentiated into a foundational phase (2000–12) marked by gradual capacity building and a subsequent acceleration phase (2013–25) characterized by rapid scaling, consistent with an emerging frontier discipline. The annual growth rate of 14.49% exceeds that typically observed in mature scientific fields, indicating sustained momentum and expanding strategic relevance. The close alignment between publication output and citation trends indicates that increased publication activity has been accompanied by increased citation activity, although whether this reflects substantive scientific advances cannot be determined from citation data alone. A pronounced acceleration between 2020 and 2025, rising from 539 to 973 publications, coincides with the COVID-19 pandemic period, although the underlying drivers of this increase fall outside the scope of publication-based analysis. Despite a brief stabilization during 2021–23, the renewed surge in 2024–25 indicates resilient long-term expansion. The relatively short citation lag further suggests that newly published studies rapidly attain visibility and influence, a pattern typical of strategically important and innovation-intensive biomedical fields.

**Figure 2:**
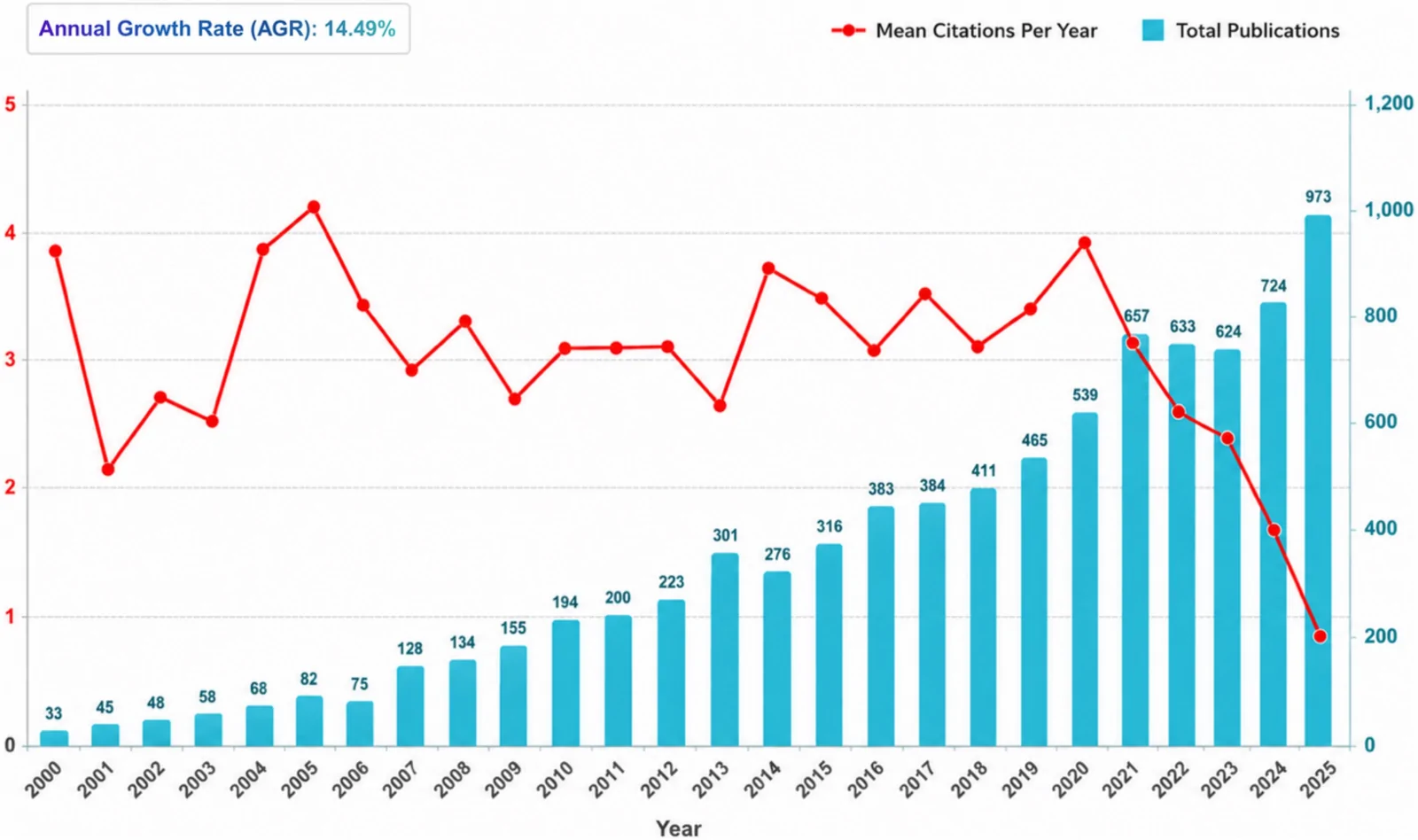
Publication volume trend and annual citations of ASEAN genomic disease surveillance research (2000–25).

Figure 3 indicates that ASEAN genomic disease surveillance research remains in a robust expansionary phase characteristic of an emerging yet not fully mature scientific field. The strong fit of observed publication data to the logistic model suggests that growth has followed a cumulative and structured trajectory rather than transient surges. The projected peak in annual output during the early 2040s represents continued modeled growth in publication volume under the fitted logistic trajectory. Because the observed data remain within the earlier segment of the cumulative S-curve, substantial scope remains for further growth in publication volume and thematic diversification, indicating that domains such as pathogen evolution, AMR genomics, zoonotic spillover surveillance, real-time outbreak analytics, and multi-omic integration are still in formative stages of publication activity. As the field approaches its future inflection point, a transition from descriptive molecular studies toward more translational publication themes, including predictive epidemic intelligence, precision public health, and integrated regional surveillance systems, may occur. Thus, the model suggests that the coming two decades will likely be important in shaping the scientific trajectory of genomic surveillance publication output in Southeast Asia, though the implications for governance and public health impact, however, require evidence beyond what bibliometric methods can provide.

**Figure 3:**
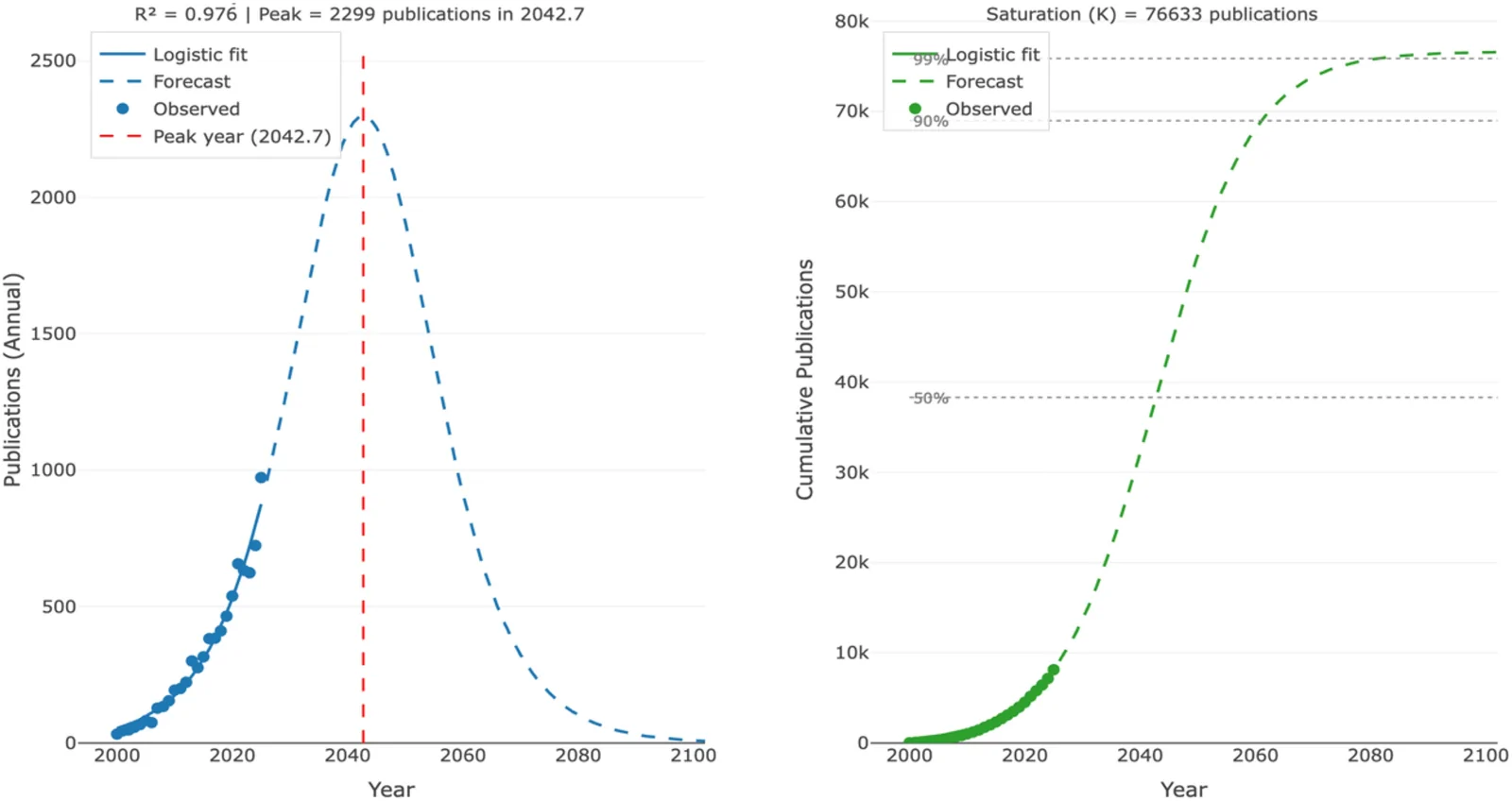
Life cycle of scientific publication and growth rate curve of ASEAN genomic disease surveillance research.

### Regional scientific productivity and collaboration network

The geospatial distribution of Scopus-indexed publications (Fig. 4) reveals a stratified landscape. Countries such as Thailand and Malaysia exhibit higher productivity per capita Gross Domestic Product (GDP) and per gross expenditure on research and development, suggesting more efficient translation of available resources into indexed scientific output. When considered alongside moderate population sizes, their output reflects not only scale but also research efficiency. In contrast, Indonesia and the Philippines show moderate output despite large populations and sizable overall GDP, implying that genomic surveillance research is more sensitive to targeted R&D intensity than to absolute economic size alone. Singapore’s high output relative to its small population aligns with its status as a high-income, research-intensive economy. Overall, the pattern reflects indexed scholarly visibility, which may underrepresent national surveillance activities that are not published in international journals or that are disseminated through local, non-Scopus channels.

**Figure 4:**
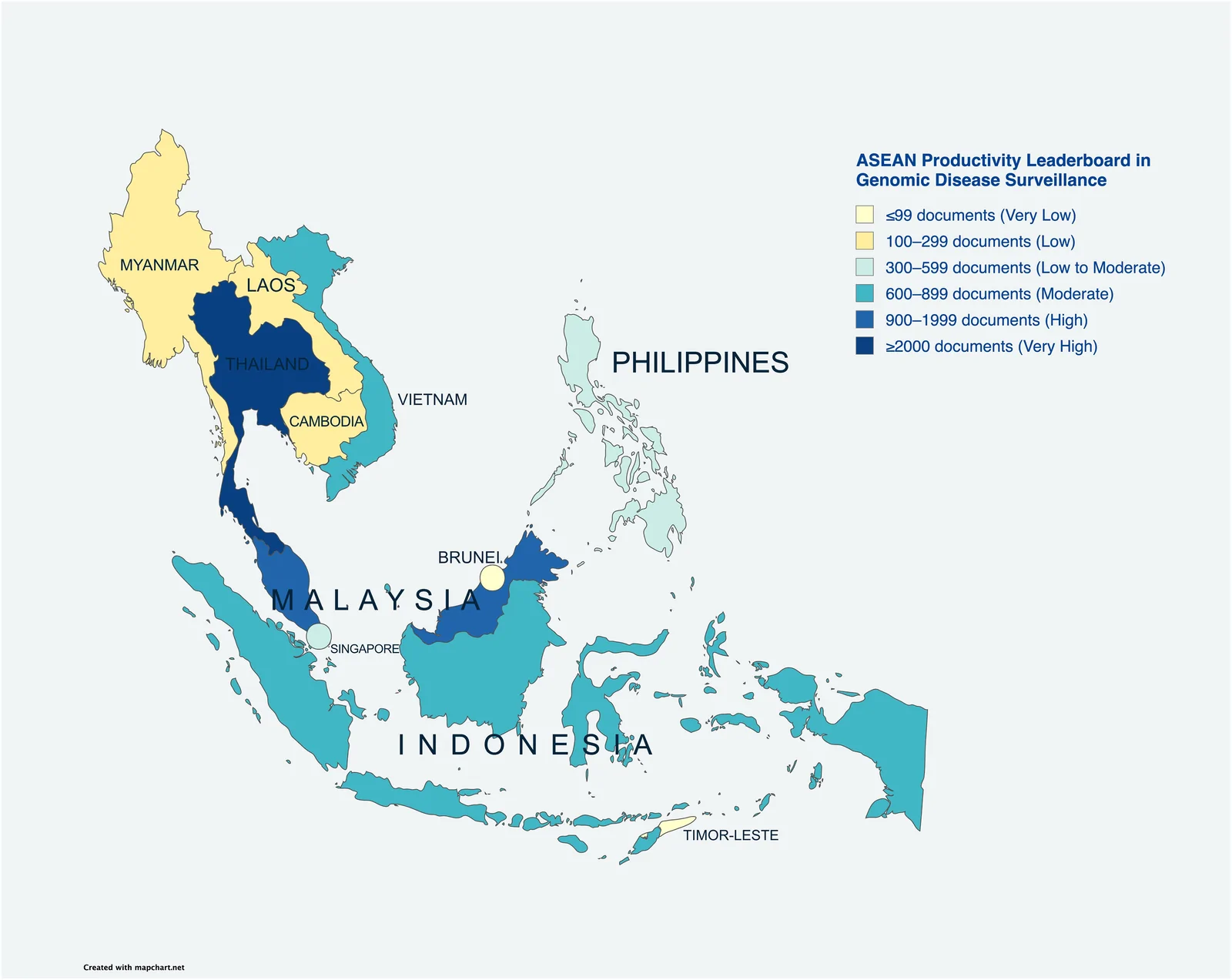
ASEAN region’s scientific productivity in genomic disease surveillance research.

The overlay visualization in Fig. 5 reveals a temporal and structural evolution of global scientific collaboration. Nodes represent countries; edge color reflects the average publication year of documents in each co-authorship link, according to the color gradient (cooler = earlier, warmer = more recent). Dense linkages surrounding the USA, several European countries, and Australia, represented by earlier cooler hues, indicate the historical dominance of high-income countries with established biotechnology infrastructures. In contrast, warmer hues across Indonesia, Malaysia, Thailand, and the Philippines during 2019–20 reflect that their collaborations with international partners have become more recent on average, consistent with growing integration into global genomic surveillance networks. Crucially, a country may have multiple edges of different colors, corresponding to collaborations with different partners at different time periods; the figure does not imply that any country published only in a single year. Indonesia’s relatively prominent recent node suggests accelerated growth, possibly associated with pandemic-driven genomic capacity building. The network indicates that middle-income ASEAN economies have become more prominent in international collaborations in recent years, although the factors underlying this shift warrant investigation through qualitative and institutional research.

**Figure 5:**
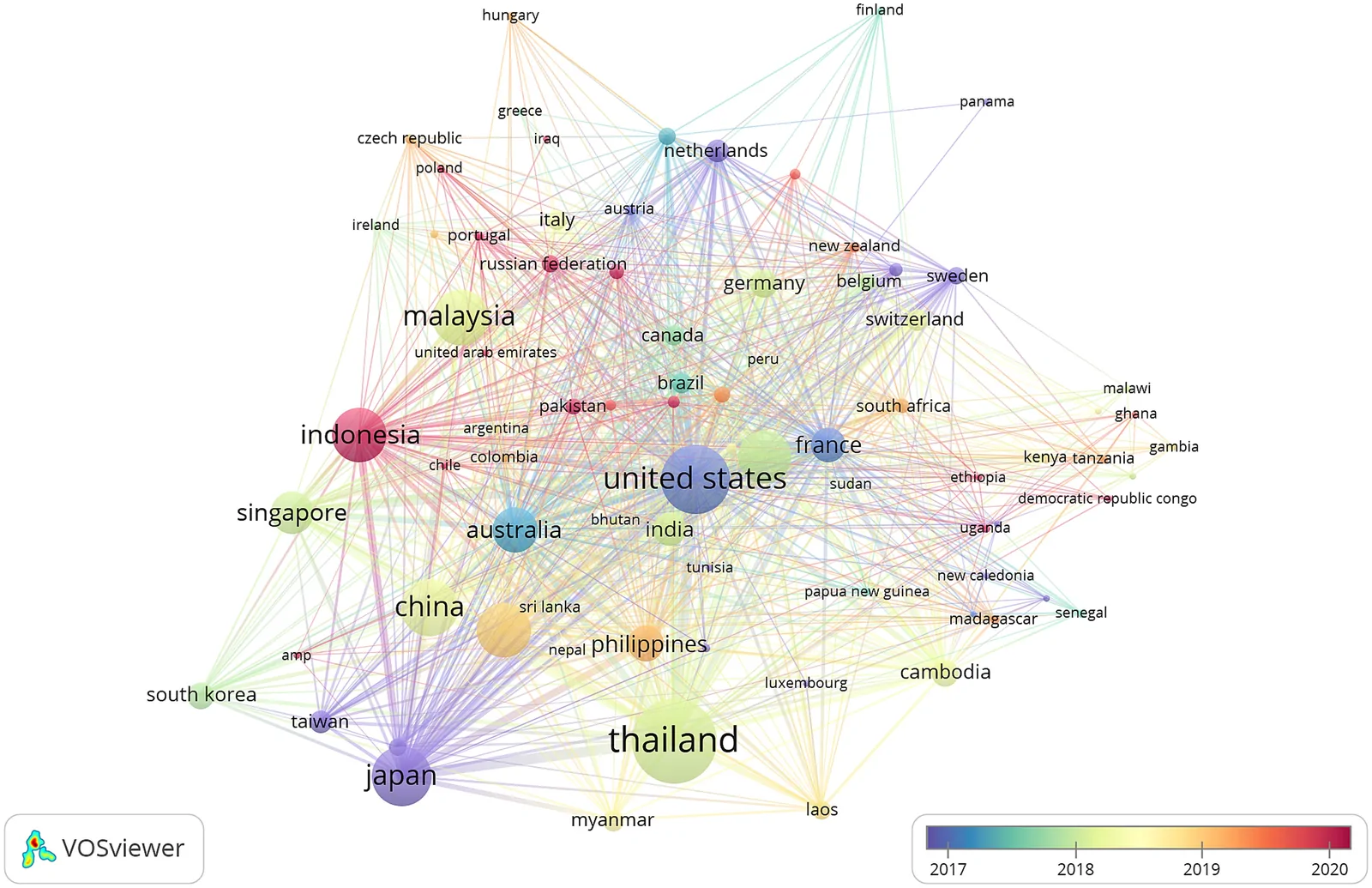
International co-authorship network in ASEAN genomic disease surveillance research through overlay visualization.

### Research funding sponsors

The funding landscape presented in Fig. 6 reveals a heterogeneous and internationally connected research-support structure, with publication activity concentrated among a relatively small group of major funding institutions. The Japan Society for the Promotion of Science was associated with the largest number of publications (388; 4.77%), followed by the National Institutes of Health (359; 4.42%), the National Institute of Allergy and Infectious Diseases (321; 3.95%), and the National Natural Science Foundation of China (282; 3.47%). Regional institutions were also prominent, particularly Chulalongkorn University (226; 2.78%), the National Research Council of Thailand (195; 2.40%), and the Thailand Research Fund (171; 2.10%). The values therefore represent the total number of publications associated with each funding agency and the corresponding percentage of its contribution to the indexed publication set, providing a comparative view of funding-associated research activity. This distribution indicates that the research landscape is substantially embedded within international and regional funding networks, with Japanese, USA, Chinese, and Thai institutions occupying prominent positions. The concentration of multiple Thai funding institutions further highlights the role of established regional research infrastructures in sustaining scientific productivity, while the presence of major international agencies reflects the importance of cross-border research support and collaboration. The funding distribution demonstrates a concentrated institutional structure of research support and highlights the presence of both domestic and international funding agencies within the regional research landscape.

**Figure 6:**
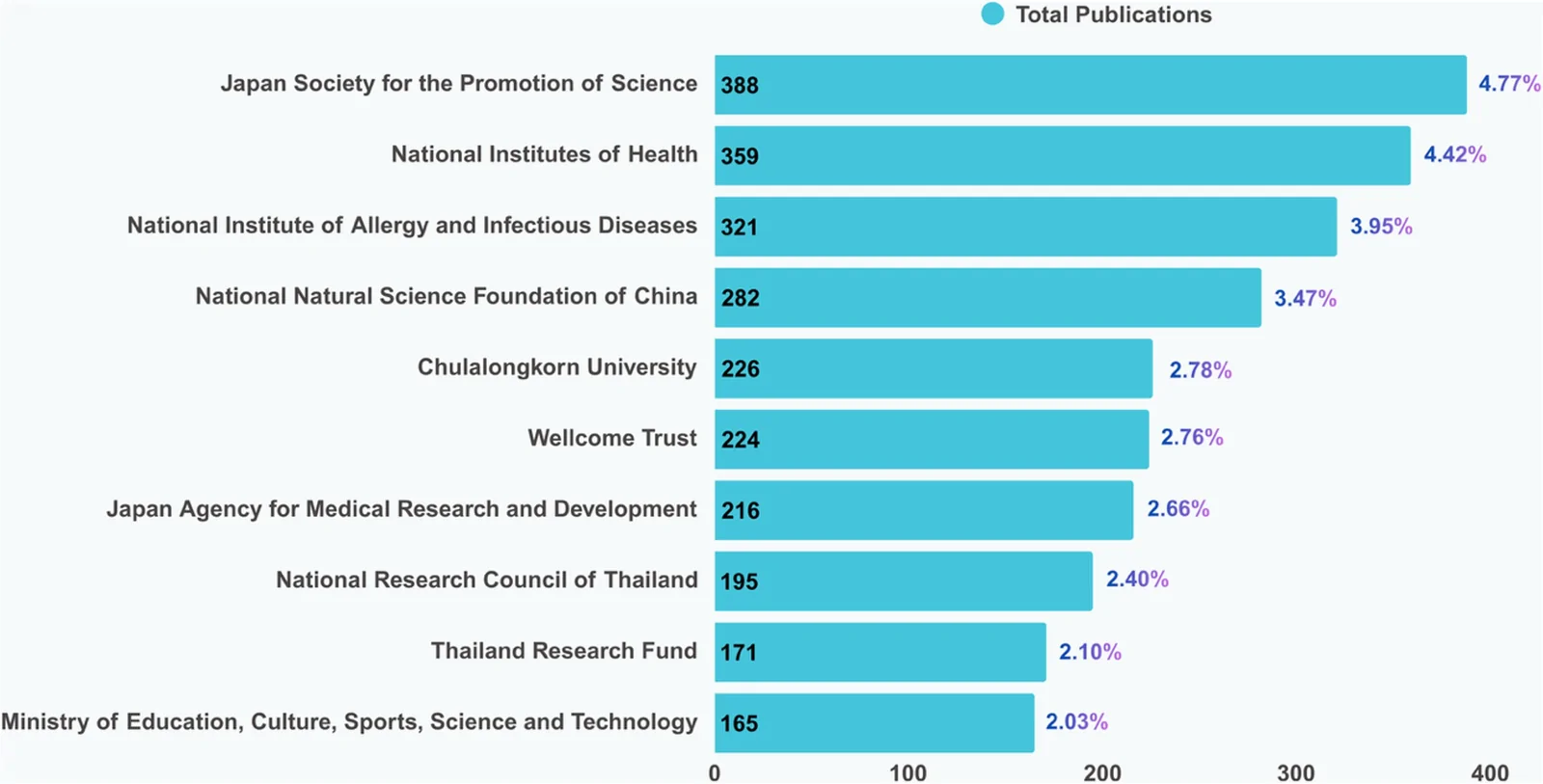
Funding agencies acknowledged in ASEAN genomic disease surveillance publications; percentages represent the proportion of indexed publications associated with each funding agency and do not represent monetary funding amounts; therefore, funding volume cannot be directly compared between countries.

### Most relevant authors and affiliations

Table 2 provides a scientometric view of the intellectual leadership and structural hierarchy of genomic disease surveillance research in ASEAN, revealing Thailand as the principal regional knowledge hub through the strong representation of Yong Poovorawan and multiple contributors from Chulalongkorn University and the Mahidol Oxford Tropical Medicine Research Unit. This concentration suggests a mature, institutionally anchored research ecosystem in which sustained investment, continuity of expertise, and embedded collaborative infrastructures have translated into high productivity and durable citation impact. The clustering of leading authors within a limited number of institutions reflects a “center of excellence” model, where well-resourced hubs drive agenda-setting, capacity building, and regional knowledge diffusion. At a finer level, the metrics distinguish productivity-based from impact-based influence: while Poovorawan’s high publication and citation counts indicate broad and sustained contributions, authors such as Arjen M. Dondorp and Tran Tinh Hien exhibit exceptionally high average citations per paper (ACP), signaling smaller but highly influential portfolios of field-defining studies. The presence of top-ranked authors affiliated with institutions in Japan, UK, and international consortia further underscores the importance of transnational collaboration, whereby global funding networks, advanced technologies, and analytical expertise intersect with local epidemiological contexts. Collectively, the findings indicate that intellectual leadership in ASEAN genomic surveillance is co-produced through institutional concentration, citation efficiency, and strategic integration within international scientific networks rather than publication volume alone.

**Table 2:** Top 10 most influential authors in ASEAN genomic disease surveillance research.

Rank	Author’s name	Affiliation	h-index	TC	TP (%)	ACP
1st	Poovorawan, Yong	Chulalongkorn University (Thailand)	38	4007	120 (1.48)	33.39
2nd	Nosten, Francois	Shoklo Malaria Research Unit (Thailand)	29	3388	45 (0.55)	75.29
3rd	Ushijima, Hiroshi	The University of Tokyo (Japan)	29	2475	89 (1.09)	27.81
4th	Theamboonlers, Apiradee	Chulalongkorn University (Thailand)	28	2038	56 (0.69)	36.39
5th	Baker, Stephen	University of London (UK)	27	2011	43 (0.53)	46.77
6th	Hien, Tran Tinh	Hospital for Tropical Diseases (Vietnam)	27	3849	34 (0.42)	113.21
7th	Maneekarn, Niwat	Chiang Mai University (Thailand)	26	2004	89 (1.09)	22.52
8th	Thwaites, Guy	Nuffield Orthopedic Center (UK)	26	1810	52 (0.64)	34.81
9th	Buchy, Philippe	Institut Pasteur du Cambodge (Cambodia)	25	2131	39 (0.48)	54.64
10th	Dondorp, Arjen M.	Mahidol Oxford Tropical Medicine Research Unit (Thailand)	25	4395	37 (0.46)	118.78

Abbreviations: ACP, average citations per paper; TC, total citations; TP, total publications, Rank was based on h-index.

**Note:** Author and institutional rankings based on h‑index and citation counts are subject to well‑known biases, including time‑dependent accumulation, field‑specific citation practices, and self‑citation effects. They reflect indexed scholarly visibility and impact within the Scopus database, not necessarily the full scientific contribution or leadership in genomic surveillance.

Figure 7 demonstrates that institutional productivity in ASEAN genomic disease surveillance is strongly hierarchical, with a small number of universities acting as the principal engines of regional knowledge production. The sustained and steep expansion of Mahidol University, followed by Chulalongkorn University, confirms Thailand’s dominant position in the regional research ecosystem, likely driven by cumulative advantages in infrastructure, sequencing capacity, skilled workforce, funding continuity, and integration into international infectious disease networks. The clear separation of these institutions from the broader cohort indicates that scientific leadership remains concentrated within highly resourced centers rather than evenly distributed across ASEAN. At the same time, the pronounced upward inflection across most institutions after 2018–20 suggests a region-wide acceleration in genomic research capacity, likely catalyzed by declining sequencing costs, wider access to bioinformatics tools, intensified preparedness investments, and the COVID-19 pandemic. Emerging catch-up trajectories from institutions such as the National University of Singapore, Universiti Malaya, Kasetsart University, and Khon Kaen University indicate a gradual diffusion of genomic capability beyond early leaders; however, persistent output gaps imply that sustained productivity depends not merely on technological access, but on long-term investment, workforce development, and durable collaborative ecosystems.

**Figure 7:**
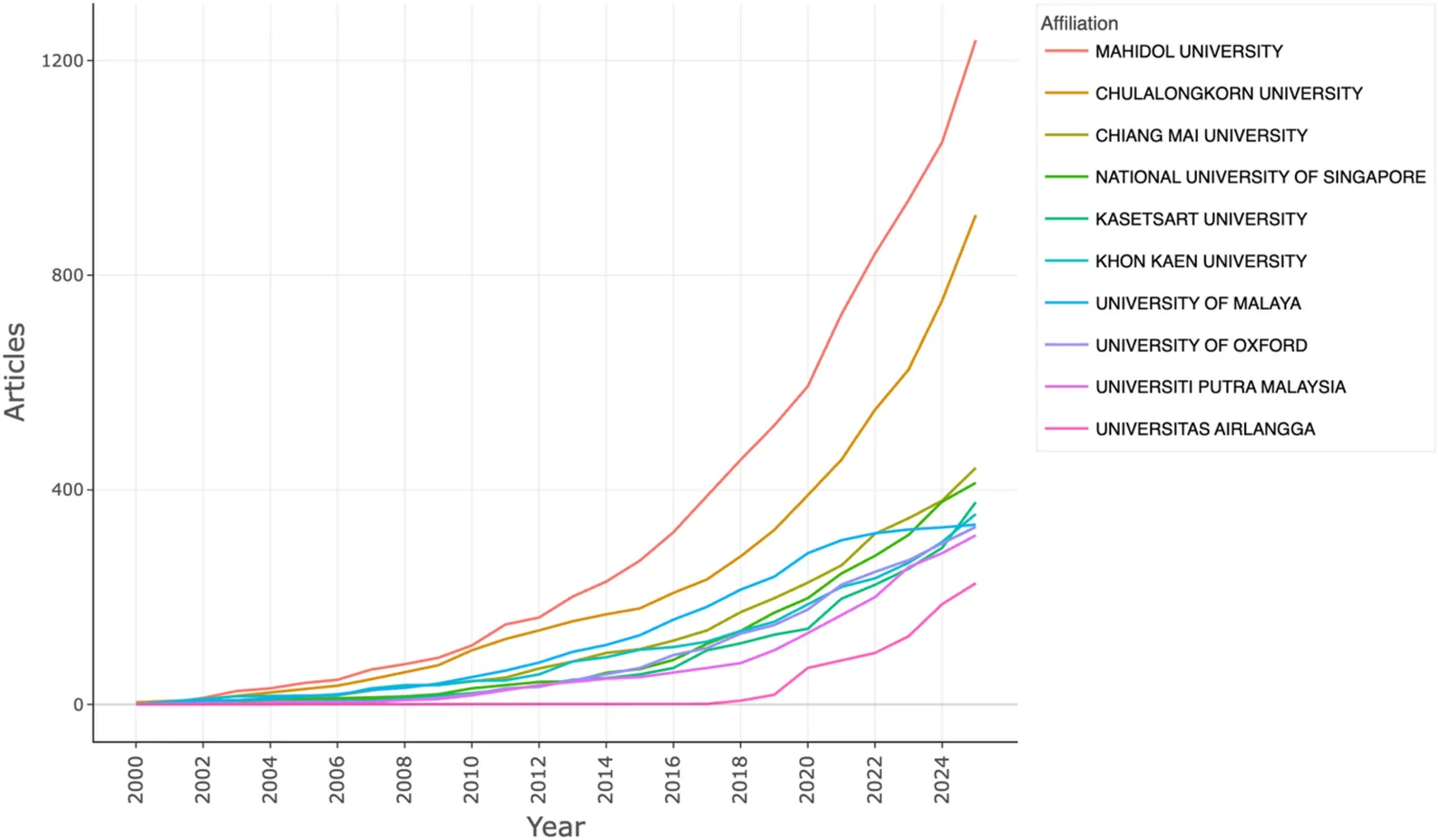
Longitudinal scientific output trajectories of the most productive academic institutions in ASEAN genomic disease surveillance research (2000–25).

### Most relevant sources

Table 3 presents a concise scientometric portrait of the publication ecosystem underpinning genomic disease surveillance research, showing that scholarly influence is concentrated within a limited group of journals that serve as key gateways for knowledge dissemination and agenda setting. The dominance of “PLOS One” in h-index (53), total citations (TC = 8955), and publication volume (TP = 287; 3.53%) indicates the strategic importance of broad-scope, open-access platforms for rapidly scaling surveillance scholarship. In contrast, the strong performance of “Emerging Infectious Diseases” and the “Journal of Clinical Microbiology” demonstrates that specialized translational journals retain substantial authority in converting genomic findings into epidemiological and diagnostic impact. Citation efficiency further distinguishes influence from output, with “Journal of Virology” (ACP = 89.24), “Journal of Clinical Microbiology” (ACP = 81.86), and “Emerging Infectious Diseases” (ACP = 67.45) producing disproportionately high-impact publications despite lower volume. The inclusion of journals spanning evolutionary biology, virology, microbiology, and tropical medicine confirms the inherently interdisciplinary character of the field, while the predominance of publishers headquartered in the USA, UK, Switzerland, and the Netherlands suggests that although much empirical surveillance work is conducted in the Global South, the principal centers of scholarly legitimacy and publication power remain concentrated in the Global North.

**Table 3:** Top 10 most influential Scopus-indexed sources publishing genomic disease surveillance research.

Rank	Journal title	h-index	Publisher (HQ)	TC	TP (%)	ACP
1st	PLOS One	53	Public Library of Science (USA)	8955	287 (3.53)	31.2
2nd	Emerging Infectious Diseases	43	Centers for Disease Control and Prevention (USA)	8431	125 (1.54)	67.45
3rd	Journal of Clinical Microbiology	43	American Society for Microbiology (USA)	6549	80 (0.98)	81.86
4th	Infection, Genetics and Evolution	37	Elsevier B.V. (Netherlands)	5218	204 (2.51)	25.58
5th	PLOS Neglected Tropical Diseases	37	Public Library of Science (USA)	4798	154 (1.89)	31.16
6th	Journal of Medical Virology	33	John Wiley and Sons Inc. (USA)	3752	147 (1.81)	25.5
7th	Frontiers in Microbiology	31	Frontiers Media S.A. (Switzerland)	2924	97 (1.18)	30.14
8th	Journal of Virology	29	American Society for Microbiology (USA)	4105	46 (0.57)	89.24
9th	Malaria Journal	29	BioMed Central Ltd (UK)	2700	96 (1.18)	28.13
10th	American Journal of Tropical Medicine and Hygiene	28	American Society of Tropical Medicine and Hygiene (USA)	8955	287 (3.53)	31.20

Abbreviations: ACP, average citations per paper; TC, total citations; TP, total publications, Rank was based on h-index.

Figure 8 illustrates a systems-level scientometric representation of how epistemic influence in ASEAN genomic disease surveillance is structurally generated through the coupling of publication venues, authorial leadership, and thematic priorities. The dense concentration of flows around a limited cohort of highly productive scholars, particularly Poovorawan, Ushijima, Maneekarn, and Theamboonlers, linked to journals such as Journal of Medical Virology, Archives of Virology, Infection, Genetics and Evolution, and Journal of Clinical Microbiology indicates that the field is organized around a concentrated author–journal nexus. This suggests that regional scientific visibility is associated with access to journals that contribute to citation accrual and dissemination within the indexed literature. Such a structure is characteristic of cumulative advantage systems, wherein established authors repeatedly occupy brokerage positions between local data generation and globally recognized publication platforms. Simultaneously, the thematic architecture remains strongly pathogen-oriented, with dominant nodes including Thailand, genotype, epidemiology, malaria, *Plasmodium falciparum*, phylogenetic analysis, and WGS, indicating that regional knowledge production has historically been shaped by endemic infectious disease burdens and country-specific surveillance infrastructures. The co-occurrence of conventional epidemiological terms with advanced genomic descriptors, together with emergent themes such as drug resistance, AMR, and COVID-19, signifies a substantive epistemic transition from organism-specific molecular characterization toward integrated, sequencing-enabled surveillance systems capable of informing real-time epidemic intelligence, transboundary risk detection, and precision public health decision-making.

**Figure 8:**
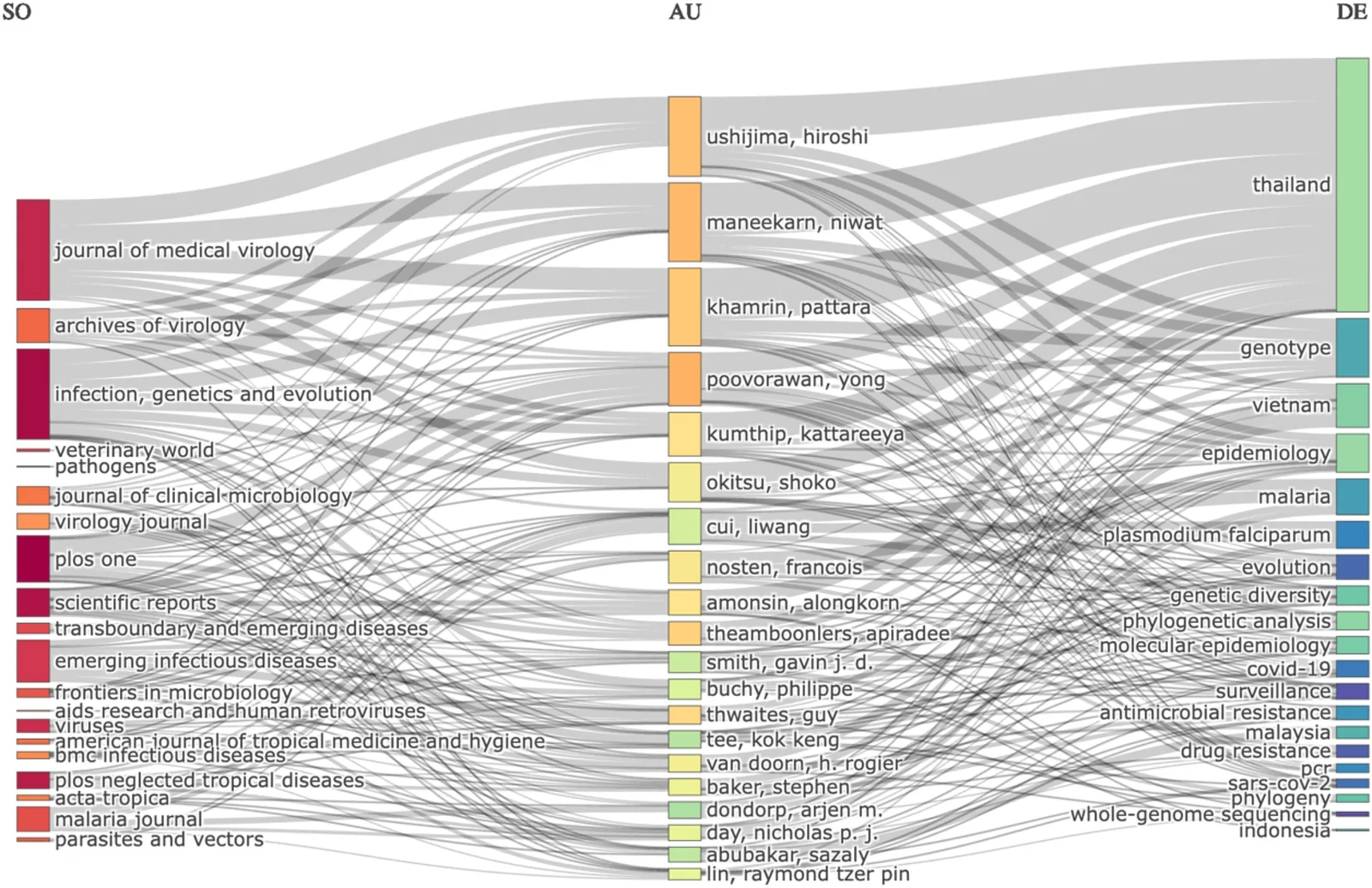
Tripartite flow analysis of sources, authors, and keywords within the ASEAN genomic disease surveillance landscape.

### Keyword co-occurrence analysis

Figure 9 reveals a densely interconnected conceptual structure in which ASEAN genomic disease surveillance is organized around multiple central knowledge clusters rather than a single dominant theme. Keywords such as phylogenetic analysis, WGS, molecular epidemiology, drug resistance, and major pathogen nodes occupy structurally central positions, indicating that genomic technologies are being operationalized not as isolated laboratory tools but as core instruments for lineage tracking, transmission reconstruction, resistance monitoring, and outbreak attribution. The simultaneous prominence of disease-specific terms related to malaria, dengue, influenza, tuberculosis, and viral infections suggests that the field has historically advanced through pathogen-priority pathways shaped by regional endemic and epidemic burdens. Overlay analysis further demonstrates a temporal shift from earlier themes centered on molecular epidemiology, phylogeny, and genetic diversity toward more recent emphases on genotype, WGS, AMR, and drug resistance, with Severe Acute Respiratory Syndrome Coronavirus 2 (SARS-CoV-2) emerging in the intermediate period. This migration implies that the pandemic functioned as a catalytic accelerant, transforming genomic surveillance from a specialized research activity into an increasingly central component of public health infrastructure. Collectively, the figure indicates an ongoing epistemic transition from fragmented organism-specific molecular studies toward an integrated, real-time, data-intensive surveillance ecosystem capable of supporting transboundary risk detection, epidemic intelligence, and precision public health across Southeast Asia.

**Figure 9:**
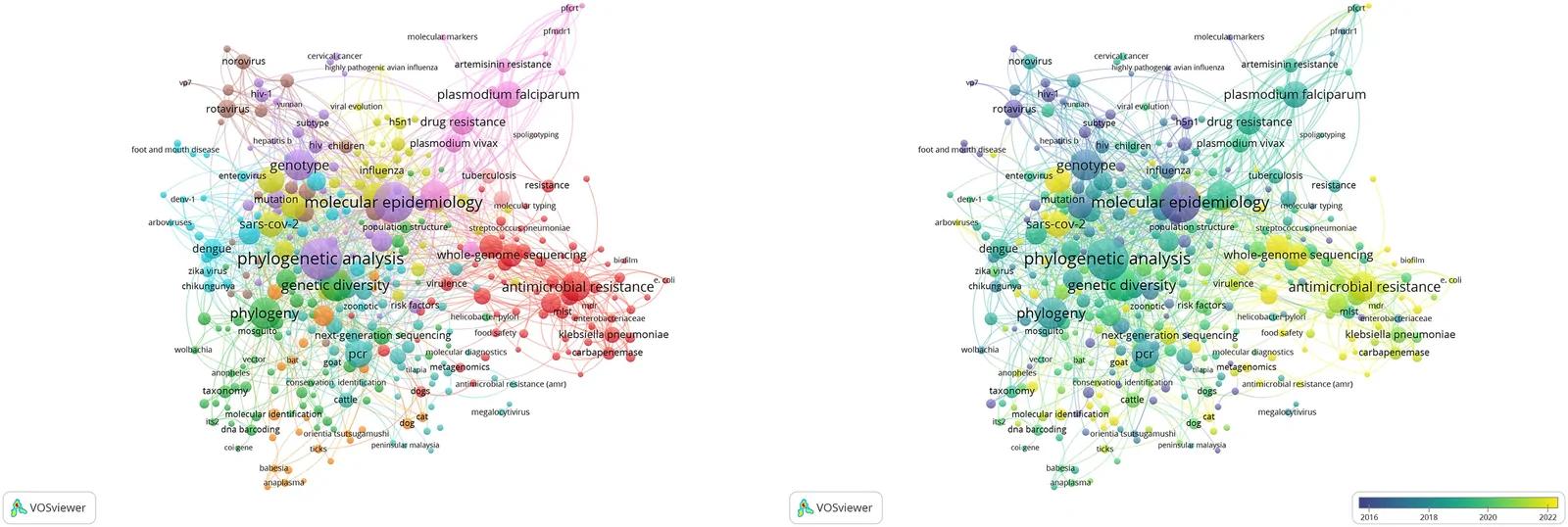
Keywords co-occurrence of ASEAN genomic disease surveillance through network and overlay analyses.

### Conceptual structure analysis

Figure 10 demonstrates that the intellectual structure of ASEAN genomic disease surveillance is undergoing a progressive transition from pathogen-specific molecular investigation toward integrated, high-resolution public health intelligence. In the thematic map, phylogenetic analysis, molecular epidemiology, genotype, and evolution occupy the motor-theme quadrant, indicating that these topics possess both high centrality and strong internal development, and therefore function as the principal methodological engines of the field. Their prominence suggests that genomic surveillance in Southeast Asia is anchored in reconstructing transmission pathways, evolutionary dynamics, and strain differentiation rather than relying solely on conventional case-count surveillance. A second strategically important cluster centered on genetic diversity, malaria, *P. falciparum*, and drug resistance reflects the enduring influence of endemic tropical disease burdens in shaping regional genomic priorities. By contrast, themes such as COVID-19, surveillance, zoonosis, and phylogenetics appear in the basic-theme quadrant, implying high systemic relevance but lower thematic consolidation; these areas likely represent rapidly expanding domains still undergoing conceptual stabilization. Meanwhile, WGS, AMR, One Health, and livestock-associated topics positioned in the emerging/declining quadrant indicate either nascent frontiers with future strategic importance or underdeveloped areas constrained by infrastructure, funding, or workforce limitations.

**Figure 10:**
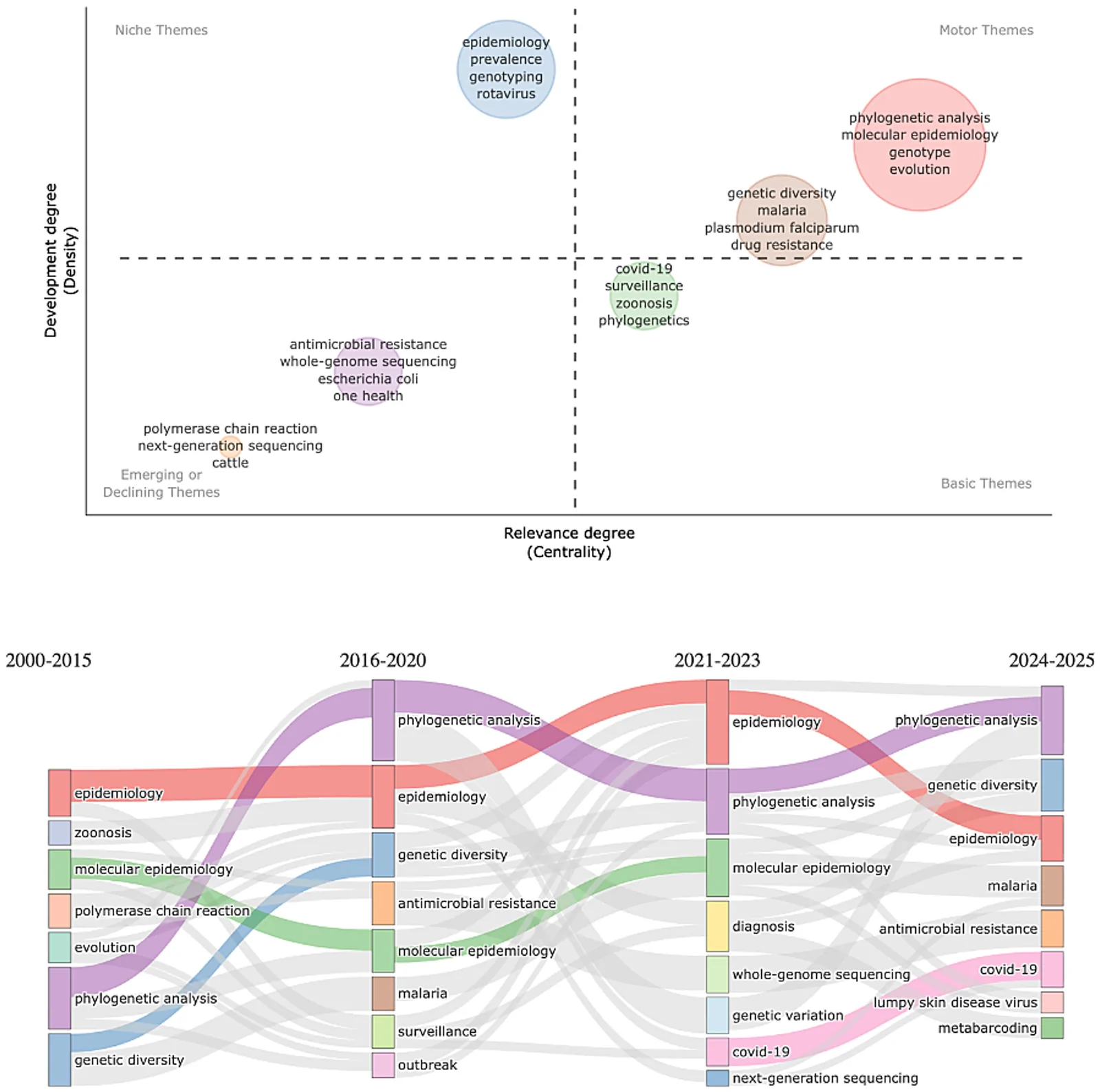
Thematic map and temporal evolution of ASEAN genomic disease surveillance research landscape.

The thematic evolution pathway further reveals a marked chronological maturation of the field across four phases. Between 2000 and 2015, the landscape was dominated by foundational descriptors such as epidemiology, molecular epidemiology, polymerase chain reaction, and phylogenetic analysis, reflecting an early era centered on diagnostic amplification technologies and descriptive molecular characterization. During 2016–20, these themes diversified into genetic diversity, AMR, surveillance, and outbreak, signifying movement from laboratory characterization toward applied population-level monitoring. The 2021–23 period marks a technological acceleration, with the emergence of WGS, genetic variation, NGS, and COVID-19, indicating that the pandemic acted as a catalytic shock that rapidly normalized advanced sequencing tools across the region. By 2024–25, the persistence of phylogenetic analysis alongside the appearance of metabarcoding, lumpy skin disease virus, and renewed attention to malaria and AMR, suggests thematic broadening beyond human viral outbreaks toward veterinary genomics, environmental biosurveillance, and cross-sector pathogen intelligence. Collectively, the figure indicates that ASEAN genomic surveillance is no longer a narrow molecular subfield, but an increasingly diversified and strategically important scientific system evolving toward precision public health, transboundary outbreak preparedness, and integrated One Health surveillance architectures.

## Discussion

This scientometric study demonstrates that genomic disease surveillance in Southeast Asia has transitioned from a niche laboratory-oriented domain into a strategically significant pillar of regional health security. The sustained annual growth rate of 14.49%, combined with the strong fit of the logistic growth model, indicates that expansion has not been episodic or opportunistic, but structurally embedded within the evolving research ecosystem, consistent with the recognized need for sustained institutional integration of genomic surveillance beyond outbreak-driven deployments [15]. In practical terms, this suggests that genomic surveillance capacity in the region is no longer dependent solely on isolated outbreak responses, but is increasingly institutionalized through universities, national laboratories, cross-border consortia, and public health agencies [16]. The marked acceleration after 2020 strongly implies that the COVID-19 pandemic served as a catalytic shock that compressed years of technological adoption into a short period, rapidly normalizing sequencing-based surveillance as an essential public health function rather than an optional research capability [17]. This mirrors global trends in which pathogen genomics has become central to variant tracking, vaccine decision-making, and outbreak forecasting [2].

A central finding of this study is the unequal geography of knowledge production across ASEAN. Thailand’s dominant presence across author rankings, institutional trajectories, and national productivity metrics suggests that it has developed the most mature genomic surveillance ecosystem in the region [18, 19]. This prominence is consistent with a model of cumulative advantages, including sustained public investment, internationally connected research institutes, and participation in multinational infectious disease networks, though bibliometric data cannot establish causality. The pattern suggests that Thailand has achieved a relatively mature research ecosystem, but direct measures of surveillance capacity, such as sequencing throughput, turnaround time, and policy integration, would be needed to confirm this. Moreover, one study found that the proportion of COVID-19 cases sequenced was significantly associated with R&D expenditure per capita, GDP per capita, and established influenza genomic surveillance capacity before the COVID-19 pandemic. In addition, 58% (72 of 124) of low-, lower-middle-, and upper-middle-income countries sequenced <0.5% of COVID-19 cases during the first 2 years of the pandemic [20]. Within Asia specifically, pathogen genomic surveillance capacity was assessed across 42 institutions in 13 South and Southeast Asian countries. Cambodia, Indonesia, Malaysia, and Thailand demonstrated more fully developed pathogen genomic surveillance capacity, while nine countries had limited system requirements to support NGS implementation for national pathogen genomic surveillance [12]. This convergence between our bibliometric results and independently assessed national capacity suggests that indexed publication output is broadly consistent with capacity assessments, though the two measures capture different dimensions of the research ecosystem.

This finding has implications for how regional research capacity is interpreted (Table 4). Our data show that indexed publication output varies substantially across ASEAN countries and should not be interpreted solely as a function of population size or aggregate economic scale. This pattern is consistent with capacity assessments in South and Southeast Asia, which have documented substantial cross-country variation in genomic surveillance infrastructure, sequencing activity, financing, workforce, and system readiness [12]. We interpret this pattern as suggesting that research visibility cannot be inferred from macroeconomic indicators alone. However, bibliometric data cannot establish whether uneven research output translates into epidemiological vulnerability or security risk. Whether countries with lower indexed output face greater outbreak risk is a question for health-systems and epidemiological research, not bibliometric analysis. We therefore present this as a pattern requiring further investigation rather than as evidence of a regional security gap. Figure 11 presents a proposed framework that synthesizes these findings, illustrating the interconnected relationships among publication growth, research capacity, regional collaboration, WGS and bioinformatics, precision public health, and next-generation epidemic intelligence in contributing to regional health security. This framework is intended as a conceptual summary rather than a validated causal model.

**Table 4:** Potential implications of scientometric findings for precision public health and epidemic intelligence.

Key scientometric finding	Potential precision public health relevance	Potential epidemic intelligence relevance
Sustained growth in genomic surveillance publications	May support wider integration of pathogen genomics into routine public health practice, subject to health-systems validation	May contribute to an evidence base relevant to outbreak monitoring, subject to operational validation
Research output concentrated in Thailand and Singapore	May reflect an uneven distribution of research activity; causal links to investment or capacity cannot be established from bibliometric data	Raises questions about the geographic distribution of surveillance infrastructure, requiring operational assessment
High international collaboration	May facilitate knowledge transfer, although the direction and equity of this transfer cannot be assessed from bibliometric data	May contribute to cross-border surveillance capacity, subject to operational validation
Shift toward WGS, AMR, and One Health	Indicates growing research attention to approaches potentially relevant to targeted public health surveillance	May be relevant to detection of emerging pathogens, contingent on laboratory and informatics capacity
Funding associated with international agencies	Raises questions about the sustainability and composition of research support; funding amounts cannot be assessed from Scopus metadata	Visibility of domestic funding agencies may be relevant to the sustainability of research support, but requires funding and health-systems research

**Figure 11:**
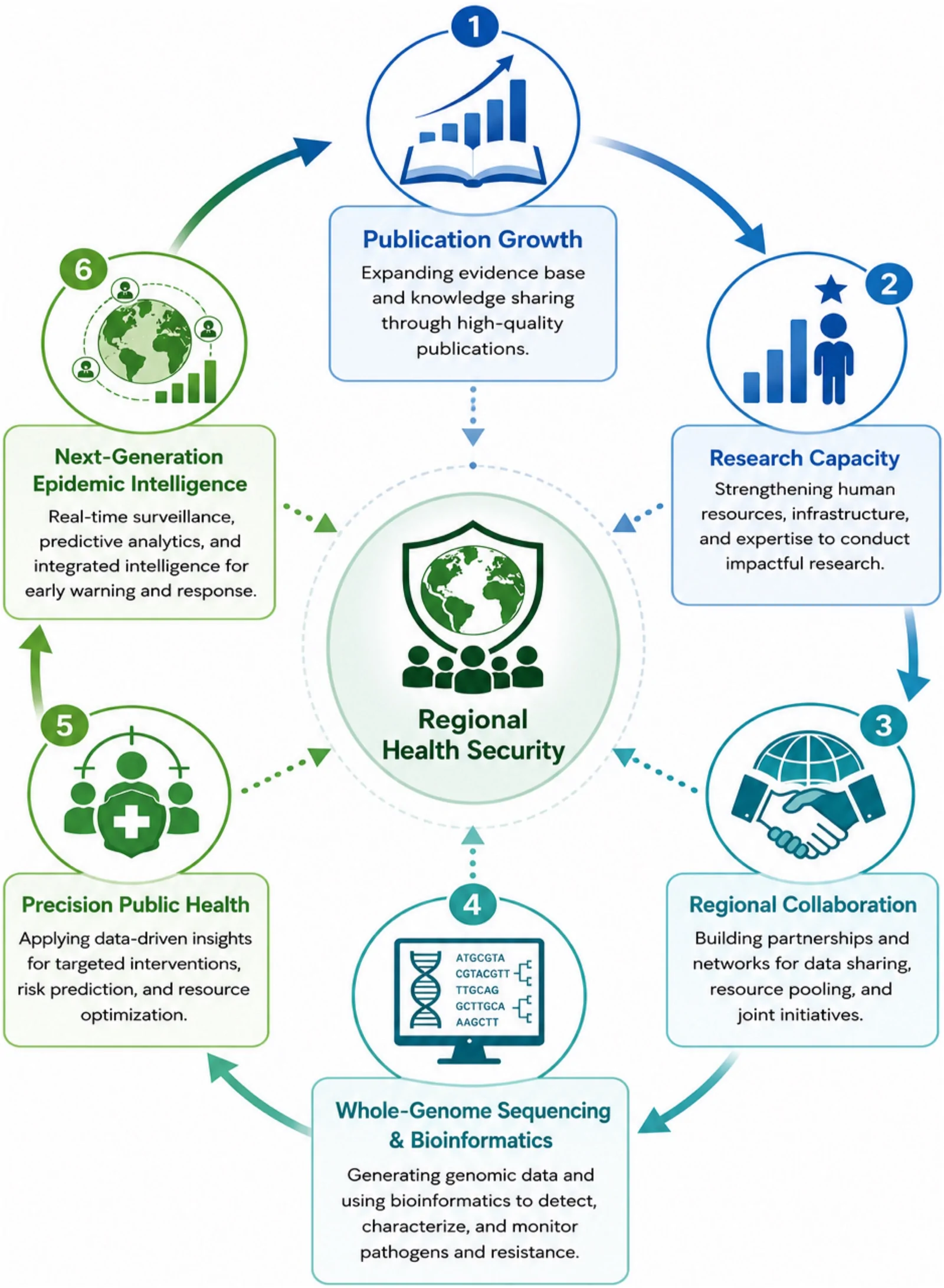
Proposed framework illustrating the progression from publication growth to regional health security; the pathway demonstrates how increasing scientific output supports research capacity and regional collaboration; this enables WGS and bioinformatics, which form the foundation for precision public health, next-generation epidemic intelligence, and ultimately, resilient regional health security.

The collaboration network further reveals that ASEAN genomic surveillance has historically been embedded within a core–periphery model dominated by high-income external partners such as the USA, UK, Australia, and Japan. This pattern mirrors the profound concentration of global infectious disease research funding in high-income countries, particularly the USA and UK [21]*.* These countries appear to have functioned as early methodological leaders, funders, and scientific brokers, shaping standards, protocols, and publication pathways. However, the more recent rise of Indonesia [22], Malaysia [23], Thailand [24], and the Philippines [12, 25] in overlay analyses indicates that Southeast Asia is progressively shifting from a peripheral sample-providing role toward more substantive participation in data generation, analysis, and agenda formation. This transition is notable because participation in genomic surveillance research involves not only sample collection but also sequencing infrastructure, bioinformatics expertise, analytical interpretation, and policy translation. Whether these capacities are equally distributed across participating countries is not resolvable through bibliometric methods and would require assessment through institutional and health-systems research.

The funding landscape reinforces this structural dependency. The strong presence of Japanese, American, British, and Chinese funding agencies demonstrates that ASEAN genomic surveillance is viewed internationally as strategically important for global health security. While such support has undoubtedly accelerated capacity building, it also raises questions regarding agenda alignment. The prominence of extra-regional funders in our dataset is consistent with findings from Africa, where genomic AMR surveillance is also heavily dependent on external donors [26], and with global analyses showing that infectious disease research funding is concentrated among funders based in high-income countries [21]. Whether this funding pattern influences research agenda-setting cannot be determined from bibliometric data alone, as Scopus funder metadata does not capture grant conditions, priority-setting processes, or the full diversity of funding sources. We therefore present this as a pattern requiring further investigation through qualitative and policy-tracing methods, rather than as evidence of donor-driven agenda distortion. Thailand’s appearance among the few countries with visible domestic funding may suggest a pathway toward greater funding autonomy, but this interpretation is tentative and would benefit from country-level case studies. Regional comparisons reinforce this structural pattern. NGS capacity in Africa remains sparse and highly concentrated, with ~71% of the continent’s 206 NGS devices located in five countries: South Africa (79), Kenya (28), Morocco (18), Nigeria (13), and Egypt (10). Most of this capacity (144 of 206 devices; 70%) was located outside national public health institutes, residing instead in academic and research facilities [27]. This pattern mirrors what we observe in ASEAN, where genomic surveillance research output is concentrated in a small number of countries and where academic institutions rather than national public health agencies are the primary drivers of research productivity. Africa has a continental coordinating body, the Africa Centers for Disease Control and Prevention, which through its pathogen genomics institute is expected to provide leadership, help develop policies and standards, and coordinate a pan-African laboratory network, while broader continental efforts are needed to strengthen data-sharing frameworks [27]. ASEAN and Africa differ in their regional coordination arrangements for pathogen genomics. Whether these differences contribute to variation in surveillance integration requires comparative institutional and health-systems research.

The publication ecosystem offers additional insight into how authority is constructed in the field. High-output journals such as “PLOS One” facilitate broad dissemination, rapid communication, and open-access visibility, all of which are valuable in surveillance sciences where timeliness matters. Yet high citation efficiency in journals such as “Journal of Virology,” “Journal of Clinical Microbiology,” and “Emerging Infectious Diseases” indicates that specialized journals disproportionately shape methodological standards and epistemic influence. This dual structure implies that genomic surveillance operates through two complementary logics: scalable dissemination and concentrated authority. This pattern suggests that publication venues may influence the visibility of Southeast Asian genomic surveillance research within the international literature.

The thematic analyses collectively indicate a major epistemic shift in the region’s research trajectory. Earlier decades were dominated by molecular typing, genotype characterization, and retrospective epidemiology [28]. These approaches were scientifically valuable but largely descriptive. Over time, the field moved toward WGS, phylogenetic analyses, AMR, COVID-19, zoonoses, and real-time surveillance architectures [29]. This transition reflects movement from static characterization toward dynamic intelligence systems capable of detecting transmission chains, monitoring evolutionary change, and informing operational decisions. Such maturation is the hallmark of precision public health, wherein interventions are targeted using fine-grained biological and spatial intelligence rather than broad reactive measures.

Equally notable is the emergence of One Health-related themes, livestock-associated pathogens, metabarcoding, and environmental surveillance. This suggests that the future of genomic surveillance in Southeast Asia will extend beyond human clinical medicine into integrated ecosystems involving veterinary health, wildlife interfaces, wastewater monitoring, vector ecology, and food systems. However, the presence of these themes in keyword networks indicates growing scholarly attention; whether this translates into operational surveillance systems requires additional empirical investigation.

Several structural considerations emerge from these patterns, though they should be read as hypotheses for further research rather than conclusions. First, the concentration of research output in a small number of institutions is consistent with a “center of excellence” model, but whether this creates operational dependency cannot be assessed through bibliometric methods. Similar concentration of sequencing capacity has been documented in Africa, where 71% of the continent’s 206 NGS devices are located in five countries and 70% of capacity resides outside national public health institutes [27], and where major hubs in South Africa, Nigeria, Kenya, Ghana, Tanzania, and Uganda account for the majority of genomic surveillance activity [26]. Second, our thematic analysis shows rapid expansion into new domains such as One Health and environmental surveillance, but bibliometric data cannot reveal whether this expansion is matched by investment in bioinformatics, data governance, or workforce retention. Third, the persistence of extra-regional funding and collaboration may have implications for intra-regional equity, but this requires investigation through funding-tracking and policy-analysis methods. Fourth, publication growth does not necessarily indicate policy uptake; comparable analyses have noted that translating genomic insights into public health action requires supportive policy, institutional, and governance frameworks [10, 26]. Each of these considerations points to specific questions for health-systems and policy research rather than definitive conclusions from our data (Table 5).

**Table 5:** Research questions arising from the scientometric findings.

Scientometric finding	What the data indicate	Question for further research
Research output concentrated in selected institutions	Research activity is unevenly distributed across institutions and countries	What factors contribute to differences in institutional research capacity across ASEAN?
Increasing publication output	Genomic disease surveillance has received increasing scholarly attention	To what extent is publication growth reflected in operational genomic surveillance capacity and public health implementation?
Emerging One Health and AMR themes	Research attention is expanding toward cross sectoral genomic surveillance topics	Are these emerging research areas being incorporated into routine surveillance and health systems?
Strong international collaboration	A substantial proportion of publications involves international co-authorship	How do international partnerships influence knowledge exchange, capacity development, and regional data sharing?
Funding associated with international agencies	International funding agencies are prominently represented in publication acknowledgements	What are the relative contributions of domestic and international funding to genomic surveillance research, and how sustainable are these funding patterns?

### Limitations

This study should be interpreted within the methodological and epistemic boundaries of scientometric analysis. The corpus represents the indexed and retrievable scholarly record, rather than the totality of genomic disease surveillance research or practice across ASEAN. Restriction to English-language Scopus-indexed publications may introduce linguistic, database, and visibility biases, particularly for locally disseminated research and non-indexed outputs. Retention of conference papers broadened coverage of emerging genomic and bioinformatics research, whereas conference reviews were excluded to maintain greater conceptual comparability with the secondary literature. Although the search strategy was iteratively refined and validated, the absence of full-text assessment across the entire corpus means that residual retrieval and classification errors cannot be completely excluded. These constraints are important because apparent differences in national or institutional productivity may partly reflect differences in publication practices, indexing coverage, and scholarly visibility rather than differences in underlying research activity.

More importantly, the unit of inference in this study is the scientific publication network, not the surveillance system itself. Publication counts, citation indicators, collaboration structures, thematic clusters, and funding associations are observational proxies of knowledge production and connectivity and should not be interpreted as direct measures of laboratory capacity, genomic testing volume, surveillance sensitivity, outbreak-detection performance, research quality, or policy implementation. Affiliation-based country attribution may also obscure multinational research arrangements, transnational data generation, and the geographic distribution of laboratory or surveillance activities, while publication-level funding acknowledgments cannot capture the full or indirect financing architecture of the field. Similarly, network and thematic associations demonstrate structural relationships in the literature but do not establish causality between funding, collaboration, research priorities, and surveillance outcomes. Accordingly, the findings provide a systematic empirical characterization of the structure, evolution, and knowledge-production dynamics of the ASEAN genomic disease surveillance literature, but extrapolation to national research capacity, health-system readiness, or real-world surveillance effectiveness should be made cautiously and, where possible, triangulated with administrative, laboratory, epidemiological, and policy evidence.

## Conclusion

Genomic disease surveillance research in Southeast Asia has experienced substantial growth, accompanied by expanding international collaboration and thematic shifts toward whole genome sequencing, AMR, zoonotic surveillance, and One Health. However, research output and scholarly visibility remain concentrated among a limited number of countries and institutions, with international funding agencies prominent in the indexed literature. These findings characterize the evolution and structure of the regional research landscape but do not directly measure surveillance capacity, health system readiness, or policy implementation. Further research integrating bibliometric, laboratory, funding, epidemiological, and policy evidence is needed to assess how research growth relates to operational genomic surveillance and precision public health across ASEAN.

**Key Points**

- Genomic disease surveillance research in Association of Southeast Asian Nations (ASEAN) has expanded rapidly, with a 14.49% annual growth rate from 2000 to 2025.
- Thailand has emerged as the principal regional knowledge hub, with research productivity and influence concentrated in leading institutions.
- Thematic evolution has shifted from molecular epidemiology toward whole-genome sequencing, antimicrobial resistance, zoonotic surveillance, and One Health.
- Research output and scholarly visibility remain concentrated in a limited number of ASEAN countries and institutions, with international funding agencies prominent in the indexed literature.

## Supplementary Material

Supplementary_Appendix_I_Search_Strategy_bbag573

## Data Availability

The dataset and all supplementary materials supporting the findings of this study, including the complete Scopus bibliographic records (8129 publications), search strategy, keyword harmonization dictionary, geographic exclusion dictionary, and analysis parameter settings for VOSviewer and Bibliometrix, are openly available in the Zenodo repository at https://doi.org/10.5281/zenodo.20822849. All data and materials are freely accessible for reproducibility and further research.
