# Supplementary material for: Mapping the frontiers of genomic disease surveillance research in the ASEAN region: scientometric insights for precision public health and next-generation epidemic intelligence
Source: Brief Bioinform. 2026 Oct 15;27(5):bbag573. doi: 10.1093/bib/bbag573 (PMC13671232; doi:10.1093/bib/bbag573)
Supplement: Supplementary file 1 [file supplementary_appendix_i_search_strategy_bbag573.pdf]

APPENDIX I

TITLE-ABS-KEY ((genomic* OR genome* OR sequencing OR "whole genome sequencing" OR WGS OR NGS OR "next generation sequencing" OR "pathogen genomics" OR "genomic epidemiology" OR "molecular epidemiology" OR phylogen* OR "molecular surveillance" OR "genetic surveillance" OR "variant tracking" OR "strain typing") AND (surveillance OR monitoring OR "disease surveillance" OR epidemiolog* OR "outbreak detection" OR biosurveillance OR "public health surveillance" OR "health surveillance" OR "event-based surveillance" OR "sentinel surveillance" OR tracking OR detection OR "transmission dynamics" OR spread OR "outbreak investigation") AND ("Southeast Asia" OR "South East Asia" OR ASEAN OR Brunei OR Cambodia OR Indonesia OR Laos OR Malaysia OR Myanmar OR Philippines OR Singapore OR Thailand OR Vietnam OR "Timor-Leste" OR "East Timor")) AND (EXCLUDE (PUBYEAR , 2026)) AND (LIMIT-TO (DOCTYPE , "ar") OR LIMIT-TO (DOCTYPE , "re") OR LIMIT-TO (DOCTYPE , "cp") OR LIMIT-TO (DOCTYPE , "ch"))
